# Tracking Upper Limb Motion via Wearable Solutions: Systematic Review of Research From 2011 to 2023

**DOI:** 10.2196/51994

**Published:** 2024-12-23

**Authors:** Eirini Karoulla, Maria Matsangidou, Fotos Frangoudes, Panayiotis Paspalides, Kleanthis Neokleous, Constantinos S Pattichis

**Affiliations:** 1 CEA-List Université Paris-Saclay Gif-sur-Yvette France; 2 CYENS - Centre of Excellence Nicosia Cyprus; 3 Department of Computer Science University of Cyprus Nicosia Cyprus; 4 Biomedical Engineering Research Centre University of Cyprus Nicosia Cyprus

**Keywords:** motion tracking, motion sensing, posture monitoring, wearable devices, upper limb rehabilitation, interactive feedback, real-time feedback, wearble technology, upper limb motion

## Abstract

**Background:**

The development of wearable solutions for tracking upper limb motion has gained research interest over the past decade. This paper provides a systematic review of related research on the type, feasibility, signal processing techniques, and feedback of wearable systems for tracking upper limb motion, mostly in rehabilitation applications, to understand and monitor human movement.

**Objective:**

The aim of this article is to investigate how wearables are used to capture upper limb functions, especially related to clinical and rehabilitation applications.

**Methods:**

A systematic literature search identified 27 relevant studies published in English from 2011 to 2023, across 4 databases: ACM Digital Library, IEEE Xplore, PubMed, and ScienceDirect. We included papers focusing on motion or posture tracking for the upper limbs, wearable devices, feedback given to end users, and systems having clinical or rehabilitation purposes. We excluded papers focusing on exoskeletons, robotics, prosthetics, orthoses, or activity recognition systems; reviews; and books.

**Results:**

The results from this research focus on wearable devices that are designed to monitor upper limb movement. More specifically, studies were divided into 2 distinct categories: clinical motion tracking (15/27, 56%) and rehabilitation (12/27, 44%), involving healthy individuals and patients, with a total of 439 participants. Among the 27 studies, the majority (19/27) used inertial measurement units to track upper limb movement or smart textiles embedded with sensors. These devices were attached to the body with straps (mostly Velcro), providing flexibility and stability. The developed wearable devices positively influenced user motivation through the provided feedback, with visual feedback being the most common owing to the high level of independence provided. Moreover, a variety of signal processing techniques, such as Kalman and Butterworth filters, were applied to ensure data accuracy. However, limitations persist and include sensor positioning, calibration, and battery life, as well as a lack of clinical data on the effectiveness of these systems. The sampling rate of the data collection ranged from 50 Hz to 2000 Hz, which notably affected data quality and battery life. In addition, several findings were inconclusive, and thus, further future research is needed to understand and improve upper limb posture to develop progressive wearable systems.

**Conclusions:**

This paper offers a comprehensive overview of wearable monitoring systems, with a focus on upper limb motion tracking and rehabilitation. It emphasizes the various types of available solutions; their efficacy, wearability, and feasibility; and proposed processing techniques. Finally, it presents robust findings regarding feedback accuracy derived from experiments and outlines potential future research directions.

## Introduction

Two out of the three leading conditions driving the need for rehabilitation are musculoskeletal and neurological disorders, with 1.71 billion and 255 million people, respectively, being affected globally. Musculoskeletal disorders have a significant impact on both individuals and society as a whole and incur a substantial economic cost. Thus, their effective and timely treatment through traditional rehabilitation approaches or digital health interventions is very important [1]. Carpal tunnel syndrome, rotator cuff tendonitis, and trigger finger are among the most common upper limb musculoskeletal dysfunctions that require methodical rehabilitation treatment [2]. People experiencing such disorders are often exposed to physical activities that require repetitive work [3].

Neurological diseases, on the other hand, not only affect the physical movement of patients but also influence their independence, living conditions, and quality of life. Usually, these disorders induce upper limb impairments, which often require conventional interventions to recover, such as physical or occupational therapy [4], as their rehabilitation depends on the duration, intensity, onset, and task orientation [5]. Stroke is the leading neurological disorder [6] and can potentially require long-term rehabilitation, with the affected person sometimes never achieving full recovery [7]. The high prevalence of stroke and the unique challenges it introduces have led to many studies about how technology can be used to assist affected populations and how to meet their needs [8].

Over the last 2 decades, technological devices have been included in rehabilitation programs within hospitals and treatment centers, which provide assistance and quantitative analysis during the rehabilitation process. Wearable devices have seen rapid development recently, with new technologies emerging constantly. The sensors used with wearables are getting smaller, more portable, and more energy efficient while, at the same time, having improved accuracy [9]. The prevention and rehabilitation of upper limb disorders are major problems that can benefit from the use of advanced wearable recovery systems. However, their integration and adoption in clinical practice are limited, and further actions are required toward this goal [6,10].

Therefore, efforts should be focused on developing systems that ensure that researchers can get reliable data for clinical evaluations. In this way, the variations in therapy and functional recovery can be better understood, aiming for the development of intervention strategies and the comprehension of the neuromuscular system [6]. Wearable devices are developed to solve these problems by providing affordable home-based solutions. It has been argued that inertial measurement units (IMUs) and surface electromyography (EMG) sensors are among the best options from the wide range of sensors currently available on the market that are used for the collection of data from wearable devices. These sensors offer a good balance between unobtrusiveness, robustness, and data quality [7].

This paper aims to provide a thorough review of how wearable-based monitoring systems are used for upper limb motion tracking and rehabilitation. More specifically, we aim to provide new insights into this area and analyze them in the following aspects: (1) assess the type and effectiveness of each wearable system; (2) assess the wearability and feasibility of the sensing technology; (3) clarify the signal processing techniques and extracted features; (4) classify the type and accuracy of the feedback according to the experiment results; and (5) review the findings, discuss limitations, and propose future directions.

## Methods

### Review Phases

The review was conducted based on the Bargas-Avila and Hornbæk approach [11] and Cochrane methodology [12], and it included 5 phases.

#### Phase 1: Potentially Relevant Publications Identified

##### Electronic Libraries

We searched 4 electronic libraries, which cover a balanced range of disciplines, including computer science and engineering, medical research, and multidisciplinary sources. The following libraries were included in the review: (1) ACM Digital Library, (2) IEEE Xplore, (3) PubMed, and (4) ScienceDirect. We restricted the search to a timeframe of 13 years (2011 to 2023).

##### Search Terms

The following queries were used: (1) “upper limb rehabilitation” AND “wearables,” and (2) (“posture monitoring” OR “motion monitoring”) AND “wearables.”

##### Search Procedure

The search terms were applied to the publication’s title, abstract, and keywords.

##### Inclusion Criteria

The inclusion criteria were as follows: (1) the paper concerns motion or posture tracking of the upper limbs, (2) the study focuses on wearable devices, (3) feedback is given to the end users, (4) the system considers clinical or rehabilitation purposes, and (5) the paper has been published in the last 13 years and is written in English.

##### Exclusion Criteria

The exclusion criteria were as follows: (1) robotic and exoskeleton systems, (2) prosthetics and orthoses, (3) activity recognition systems (activity/gesture or motion capture), and (4) reviews and books.

##### Search Results

The total number of search results from phase 1 was 1564 papers. More detailed results are presented in Table 1.

**Table 1:** Search results per library.

Query	Library (N=1564), n				
	ACM^a^	IEEE^b^	PM^c^	SD^d^	Total
Query 1^e^	338	145	309	78	870
Query 2^f^	228	99	67	300	694

^a^ACM: ACM Digital Library.

^b^IEEE: IEEE Xplore.

^c^PM: PubMed.

^d^SD: ScienceDirect.

^e^Query 1: “upper limb rehabilitation” AND “wearables.”

^f^Query 2: (“posture monitoring” OR “motion monitoring”) AND “wearables.”

#### Phase 2: Publications Retrieved for Detailed Evaluation

##### First Exclusion

All 1564 search results from phase 1 were imported into the software “Paperpile.” Duplicate entities were excluded manually. Overall, 96 duplicate publications were removed, and 1468 papers remained.

##### Second Exclusion

Publications with incomplete or restricted entries, those with no available full text, and those considered irrelevant based on the abstract were excluded manually. As a result, 1110 papers were removed.

##### Third Exclusion

We narrowed the entries to original full papers written in English. Part of this third exclusion was to remove entities that were not original full papers, such as workshops, posters, speeches, reviews, magazine articles, and generally grey literature without formal peer review. As a result, 51 papers were excluded. The remaining 307 papers included 255 journal articles, 45 conference papers, and 7 book chapters. 

#### Phase 3: Publications to be Included in the Analysis

##### Final Exclusion

The focus of this review is on tracking upper limb motion via wearable solutions. Consequently, this final exclusion phase excluded studies that were not relevant based on a full-text review. Based on the exclusion criteria, we removed 280 irrelevant publications (eg, exoskeleton, robotics, and orthosis), and finally, 27 papers were selected for analysis.

##### Phase 4: Data Gathering

The screening process and data extraction were performed independently by 3 researchers (EK, MM, and PP). Discrepancies between reviewers were resolved through regular meetings and detailed discussions, addressing all the disagreements to minimize bias. In this phase, relevant information was extracted from the selected papers to conduct the analysis. From each study, the following information was extracted: target population, sample size of participants, sensor placement, type of the wearable system, type and feasibility of wearability, data sampling rate, energy consumption/battery characteristics, type and accuracy of feedback according to statistical analysis results, methodology, measurement techniques, instruments, key findings, limitations, and future directions.

##### Phase 5: Data Analysis 

The data collected in phase 4 were analyzed using descriptive statistics. We then reviewed the literature to support and enhance the additional knowledge that this paper provides. Thematic analysis was used as an extra methodology to categorize our findings based on themes: (1) type and effectiveness of wearable systems, (2) wearability of sensing technology, (3) data processing and measurement techniques, and (4) type and accuracy of feedback.

## Results

### Study Characteristics

**Figure 1:**
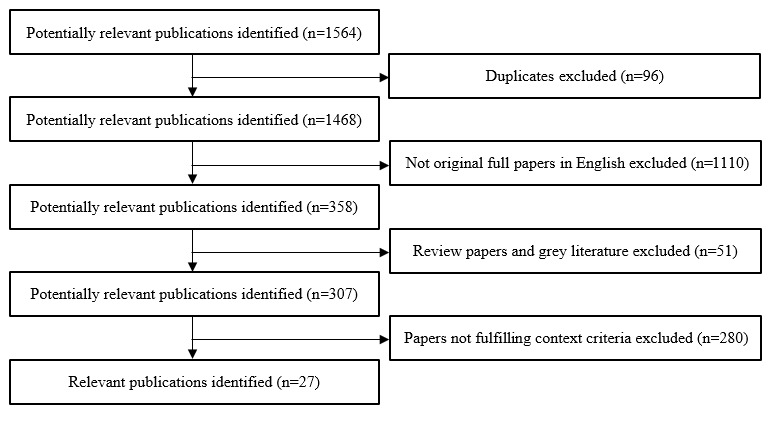
Identification and selection flow diagram.

The review identified 27 studies (Figure 1). All the reviewed studies tracked upper limb motion using wearable devices and were divided into 2 main categories according to the purpose of the study: (1) clinical motion tracking and (2) rehabilitation. Some studies examined both applications. More specifically, the majority of papers that were selected (15/27, 56%) [13-27] focused on wearable devices that monitor human movement, and data were collected to be employed for general use in a clinical setting. The remaining papers (12/27, 44%) [28-39] emphasized systems for upper limb rehabilitation. The PRISMA (Preferred Reporting Items for Systematic Reviews and Meta-Analyses) checklist is provided in Multimedia Appendix 1.

The key features of the studies are summarized and compared in Table 2, according to the purpose of the study, the type and number of sensors used, the placement location of the sensors, the placement method for the sensors, and the measurement method, with division into the 2 aforementioned categories.

**Table 2:** Key features of the studies.

Content and study			Purpose		Technology (number of sensors)		Placement location		Placement method		Measurement/methodology
**Motion tracking**											
	Yu et al [27]	Monitor upper limb motion function of stroke patients		Accelerometer (n=2)		Forearm, upper arm		Straps		ROM^a^: Bobath’s handshake and shoulder touch	
	Wang et al [24]	Monitor compensatory movements and evaluate their applicability in a clinical setting		9-DOF^b^ IMU^c^ (n=2)		Shoulder, torso		Zipped vest, Velcro straps		Compensatory movement of the shoulder girdle	
	Li et al [18]	Evaluate upper limb motor function in hemiplegic patients		6-DOF IMU (n=2), EMG^d^ (n=10)		Wrist, forearm, upper arm		Wristband, armband		Movements of major upper limb joints: shoulder, elbow, wrist, and finger joints	
	Repnik et al [20]	Quantify upper limb movement for muscle activity analysis in stroke patients		9-DOF IMU (n=7), EMG (n=2)		Hand, wrist, forearm, upper arm, sternum		Wristband, armband, straps		Movement quantified: hand smoothness, trajectory, trunk stability, and muscle activity	
	Tolvanen et al [23]	General motion tracking		Piezoresistive strain sensor (n=1)		Hand, wrist, forearm, upper arm (biceps, triceps)		Reusable adhesive layer		Opening-closing cycles (hand), muscle tension of the flexor sternum, biceps, upper arm, bicep curl, peck fly, and triceps	
	Bai et al [13]	Monitor different body movements, muscle contraction, and relaxation		GYS^e^ sensor		Upper arm, fingers		Direct winding		ROM: instant tensing, bending, static motions of fingers, varied contractions of the bicep	
	Gu et al [15]	Identify hand motions, joint bending, hand posture, gesture, and sign language		Hydrogel-elastomer hybrid ionic sensor (n=10)		Hand, fingers		Water-borne adhesive		ROM: finger bending/extending and hand gestures	
	Lee et al [17]	Analyze data from neurologically intact individuals and the free-living environment, and develop a system to monitor stroke survivors		Accelerometer (n=4)		Wrists, fingers		Rings, wristbands		General quantification of the amount of use of upper limb function	
	Zhang P et al [26]	Monitor human movement with the use of a flexible resistance strain sensor with a porous structure		Strain sensor (n=1)		Upper arm, forearm, wrist, fingers		Velcro straps		ROM: finger wrist and elbow bending; responses to breathing	
	Lee et al [16]	Facilitate the clinically fitted measurement of fine-motor finger and wrist joint movements. Characterize age-related changes in hand functions		9-DOF IMU (n=7)		Wrist, hand, fingers		Clip-on straps		ROM: finger movement (index finger, thumb flexion/extension) and wrist movement (ulnar/radial flexion)	
	Zhang J et al [25]	Implement 3D motion velocity measurement, and propose a functional link artificial neural network model (FLANN)		Microthermal flow sensor (n=2)		Wrist		Straps		Trunk velocity, relative limb velocity, and absolute limb velocity	
	Schwarz et al [21]	Evaluate spatiotemporal kinematic metrics for the assessment of upper limb movements after stroke		6-DOF IMU (n=8)		Sternum, shoulder, upper arm, forearm, hand, fingers, thumb		Medical tape/3D-printed flexible straps		ROM: shoulder, elbow, thumb, index flexion/extension, and wrist supination/pronation	
	Formstone et al [14]	Develop a comprehensive system designed for the clinical environment, and quantify hand/wrist movement		9-DOF IMU (n=3), MMG^f^ (n=2)		Sternum, upper arm, wrist, forearm		3D-printed housing cases attached with 3D-printed flexible resin straps		Shoulder twist angle (range), abduction, flexion, elbow twist angle, wrist flexion, and circumduction muscle activity	
	Little et al [19]	Analyze kinematic and physiological features for predicting elbow motion intention		9-DOF IMU (n=3), EMG (n=4), stretch sensor (n=1)		Forearm, upper arm, torso		Straps, direct winding		Muscle activity, elbow flexion angle, and custom-made changes in muscle volume	
	Schwerz de Lucena et al [22]	Real-time quantification of the effect of wearable feedback on hand counts for increasing hand activity		6-DOF IMU (n=1), magnetometer (n=4)		Wrist, fingers		Wristband, ring		“Hand counts”: finger flexion/extension, wrist flexion/extension, and wrist radial/ulnar deviation movement	
**Rehabilitation**											
	Ding et al [39]	Measure orientation and correct arm posture using vibrotactile actuators for stroke rehabilitation patients and therapists		9-DOF IMU (n=2)		Forearm, upper arm		Velcro straps		Body segment posture; forearm and upper arm orientation; trajectory of upper arm’s yaw, pitch, and roll; elbow angle; and forearm roll	
	Kim et al [31]	Wearable upper limb motion tracking method for stroke rehabilitation therapy at home		6-DOF IMU (n=2)		Wrist, upper arm		Velcro straps		ROM: position and orientation of the wrist and elbow joints. Accuracy of motion estimation and motion matching	
	Mohammadzadeh et al [34]	Develop and evaluate the feasibility of a wearable sensor-based motion-tracking system		6-DOF IMU (n=3)		Forearm, upper arm, sternum		Velcro straps		ROM: elbow joint angle	
	Ploderer et al [35]	Patient monitoring system to support occupational therapists in upper limb rehabilitation work with stroke patients		9-DOF IMU (n=3)		Shoulder, upper arm, wrist		Velcro straps, medical tape		ROM of each degree of freedom	
	Wang et al [28]	Evaluate garments equipped with sensors that support posture monitoring; used in upper extremity rehabilitation training of stroke patients		9-DOF IMU (n=3)		Scapula (shoulder blade), torso		Vest with Velcro straps		Analytical shoulder flexion, and analytical and functional elevation in the scapular plane	
	Salchow-Hömmen et al [37]	Part of a feedback-controlled hand neuroprosthesis for the rehabilitation of patients who experience motor impairment of the hand		9-DOF IMU (n=16)		Hand, fingers, forearm		Skin-friendly tape		ROM: combined abduction and flexion motion	
	Semjonova et al [38]	Evaluate the impact of the Double Aid (DAid) smart shirt; training process of patients with subacromial pain syndrome		Strain sensors (n=2)		Scapula		Commercial elastane-based fitness shirt		Perform exercise without moving the shoulders; detect movement or no movement of the shoulders	
	Friedman et al [29]	Nonobtrusive option for monitoring wrist and hand movement; needed for stroke rehabilitation and other applications		Triaxial magnetometer (n=2), accelerometer (n=1)		Wrist, fingers		Watch-like enclosure, small neodymium ring worn on the index finger		Accuracy of monitoring finger motion, wrist flexion/extension, and wrist ulnar/radial deviation. Accuracy in estimating different levels of movement activity	
	Kortier et al [32]	Ambulatory system using inertial sensors for hand kinematics, and evaluation of hand functioning		6-DOF IMU (n=15), 9-DOF IMU (n=6)		Hand, fingers, thumb		Double-sided adhesive tape/mounted on polyamide/elastane-fabricated glove		Static accuracy (ROM: flexion/extension, dynamic range, and repeatability)	
	Kim et al [30]	Identify optimal sensor locations		Bending sensor (n=2)		Thumb, hand		Flexible fabric straps partially made of Lycra sewed on a glove structure		ROM; circumduction motion	
	Liu et al [33]	Primary use of conductive stretchable fabrics to sense skin deformation during joint motion and infer the joint rotational angle		Strain sensor (n=2)		Forearm		Fabric		ROM: elbow flexion by various degrees; repeat motion at 3 levels of speed. Repeat each motion and perform free-form motions	
	Pregnolato et al [36]	Define the clinical features of stroke patients while performing hand movements for rehabilitation training		9-DOF IMU (n=1), EMG (n=1)		Forearm		Armband		Detect the total muscle activity of the forearm circumference	

^a^ROM: range of motion.

^b^DOF: degrees of freedom.

^c^IMU: inertial measurement unit.

^d^EMG: electromyography.

^e^GYS: graphene thin-film yarn sensor.

^f^MMG: mechanomyography.

### Study Type and Effectiveness

As mentioned above, wearable devices are used to capture upper limb function for general health purposes to obtain information and data about physiological parameters to assist rehabilitation patients and therapists. The experiments in most of the studies (22/27) [13-19,21,22,24-26,28,29,31-37,39] were noncontrolled experiments, where participants were either healthy able-bodied individuals or patients who experienced upper limb disorder, and they were recruited for participation in the experimental evaluation. In some studies (4/27) [20,27,30,38], the experiments were controlled, where both patients and healthy participants were included and the results were compared statistically. The number of participants was not specified in 1 study [23].

The findings revealed that most of the studies (22/27) [13-19,23-29,31,32,34-39] had a positive outcome. In 2 cases [22,30], further studies need to be conducted. In the first study, the maximum estimated error exceeded the required accuracy for a typical clinical assessment (over 5 degrees). In the second study, the hand feedback for stroke patients was briefly modifiable, indicating no therapeutic benefit over a short period. Thus, these studies are considered neutral. The outcomes of the remaining 3 studies [20,21,33] were negative as they do not offer any major findings [20] on motion tracking and the application is difficult to implement [21,33] due to limitations such as the system’s measurement accuracy and large errors.

From the above information, it can be concluded that motion-tracking wearable devices have an overall positive impact on the interpretation of the data, and they provide useful assessments that can be used in future systems for clinical and rehabilitation purposes. The aforementioned data are presented in Table 3, categorized according to the purpose of the study.

**Table 3:** Study type and target population.

Content and study			Study type		Target population	
**Motion tracking**						
	Yu et al [27]	Controlled		23 stroke patients, 4 physicians		
	Wang et al [24]	Noncontrolled		8 musculoskeletal shoulder pain patients		
	Li et al [18]	Noncontrolled		16 healthy adults		
	Repnik et al [20]	Controlled		28 stroke patients, 14 healthy adults		
	Tolvanen et al [23]	Not specified		Not specified		
	Bai et al [13]	Noncontrolled		1 healthy adult		
	Gu et al [15]	Noncontrolled		1 healthy adult		
	Lee et al [17]	Noncontrolled		35 healthy adults		
	Zhang P et al [26]	Noncontrolled		1 healthy adult		
	Lee et al [16]	Noncontrolled		34 healthy adults		
	Zhang J et al [25]	Noncontrolled		1 healthy adult		
	Schwarz et al [21]	Noncontrolled		9 stroke patients		
	Formstone et al [14]	Noncontrolled		3 healthy adults		
	Little et al [19]	Noncontrolled		3 healthy adults		
	Schwerz de Lucena et al [22]	Noncontrolled		20 chronic stroke patients		
**Rehabilitation**						
	Ding et al [39]	Noncontrolled		5 healthy adults		
	Kim et al [31]	Noncontrolled		4 healthy adults		
	Mohammadzadeh et al [34]	Noncontrolled		8 healthy adults		
	Ploderer et al [35]	Noncontrolled		1st study: 8 occupational therapists; 2nd study: 1 healthy participant; 3rd study: 2 occupational therapists		
	Salchow-Hömmen et al [37]	Noncontrolled		4 healthy adults		
	Semjonova et al [38]	Controlled		17 primary subacromial pain syndrome patients and 17 healthy adults		
	Wang et al [28]	Noncontrolled		17 stroke patients		
	Friedman et al [29]	Noncontrolled		7 healthy adults		
	Kortier et al [32]	Noncontrolled		1st study: 1 participant; 2nd study: 1 participant; 3rd study: 5 participants		
	Kim et al [30]	Noncontrolled		10 participants for the optimal sensor location and 4 participants for experimental evaluation		
	Liu et al [33]	Noncontrolled		10 healthy adults		
	Pregnolato et al [36]	Noncontrolled		117 stroke adults		

### Wearability and Feasibility of Sensing Technologies

#### Categorization of Wearable Sensors

Capturing the motion of the upper limbs through sensor technologies has been essential for the development of interactive wearable devices for rehabilitation and clinical setting purposes. From the reviewed papers, 4 main categories of sensing technologies were identified: (1) inertial-based sensors (accelerometer, magnetometer, and IMU); (2) bending, force, and strain sensors (bending/stretch sensor and strain sensor [piezoresistive strain sensor and hydrogel-elastomer ionic sensor]); (3) myography sensors (EMG and mechanomyography [MMG]); and (4) other sensors (graphene thin-film yarn sensor [GYS] and microthermal flow sensor).

**Figure 2:**
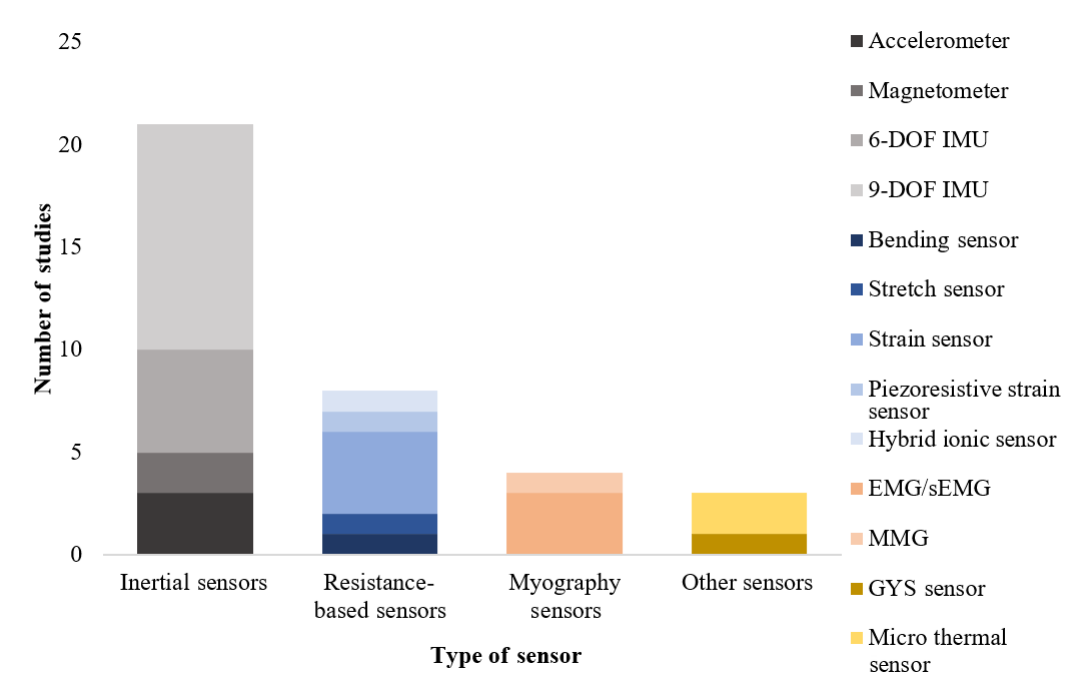
Categorization of sensing technology. DOF: degrees of freedom; EMG: electromyography; GYS: graphene thin-film yarn sensor; IMU: inertial measurement unit; MMG: mechanomyography.

Figure 2 provides an overview of the number of sensing technologies that were used in the studies, according to the type of sensor.

As Figure 2 shows, inertial sensors are the most common type of sensor used for data acquisition (19/27) [14,16-22,24,27-29,31,32,34-37,39]. An inertial sensor is an electronic device that measures the force and the angular rate of a body, which can be achieved by a combination of 3 embedded sensors: accelerometer, gyroscope, and magnetometer. It can also be used to calculate the orientation of the body. The accelerometer measures the proper acceleration, the gyroscope is used for measuring orientation and angular velocity, and finally, the magnetometer measures the strength and sometimes the direction of the magnetic field. Of the 19 studies, 14 [16,17,21,24,27-29,31,32,34,35,37-39] used a combination of the 3 sensors to measure upper limb posture. More specifically, in 6 studies [16,24,28,35,37,39], 9 degrees of freedom (DOF) inertial sensors were used, including a 3-axis accelerometer, a 3-axis gyroscope, and a 3-axis magnetometer. In 3 studies [21,31,34], the magnetometer was excluded from the data fusion, and 6-DOF inertial sensors were proposed. Two of the studies [17,27] used only accelerometers as their sensing technology, while in 1 study [29], only a magnetometer was included. Moreover, a combination of 6-DOF and 9-DOF IMUs was used [32] to measure finger movement for the assessment of hand kinematics while using inertial sensors. Another way to capture wrist and finger motion was introduced by Schwerz de Lucena et al [22], where magnetometers were placed on the wrist to capture the magnetic field changes of the index finger, and the orientation of the wrist was quantified by a 6-DOF IMU. Furthermore, Pregnolato et al [36] used a combination of a 9-DOF IMU with EMG. Only the gyroscope of the inertial sensor was used to place the device on the patient’s forearm, and EMG was used to detect the overall muscle activity in the circumference. The remaining 4 studies [14,18-20], proposed a fusion of inertial sensors with EMG or MMG [14,18,20] and a stretch sensor [19] to measure myographic data and changes in muscle volume, respectively.

In addition, the use of bending sensors and strain sensors was proposed in 2 papers [30,38]. This type of sensor was introduced owing to the limited available information on the actual impact of smart garments on clinical outcomes in physiotherapy [38] and the inaccurate measurement of thumb carpometacarpal joint movements [30]. Semjonova et al [38] proposed a purely textile-based smart shirt for the training process of patients with subacromial pain syndrome, while another study [30] presented a novel approach to identify optimal sensor locations to properly measure carpometacarpal joint configurations. Low-cost everyday fabrics were also introduced [33], which consist of stretchable conductive fabrics as strain sensors to sense skin deformations during elbow joint motion and infer the joint rotation angle. Furthermore, 1 study [23] proposed strain sensors along with a highly functional piezoresistive strain sensor, which was designed and fabricated exclusively because it provides excellent durability in human motion monitoring. Strain [26] and hydrogel-elastomer ionic [15] sensors are also used as they provide flexibility when they are worn, allowing the precise monitoring of upper limb movement and respiratory changes.

An alternative approach was also provided [13,25] as microthermal flow sensors and GYSs were introduced. The former method calculates velocity without integral calculation, and thus, accumulated errors are excluded. In the latter, the degree of resistance recovery and the gauge sensitivity can be well controlled and modulated, providing high evaluability for developing next-generation wearable electronics.

**Figure 3:**
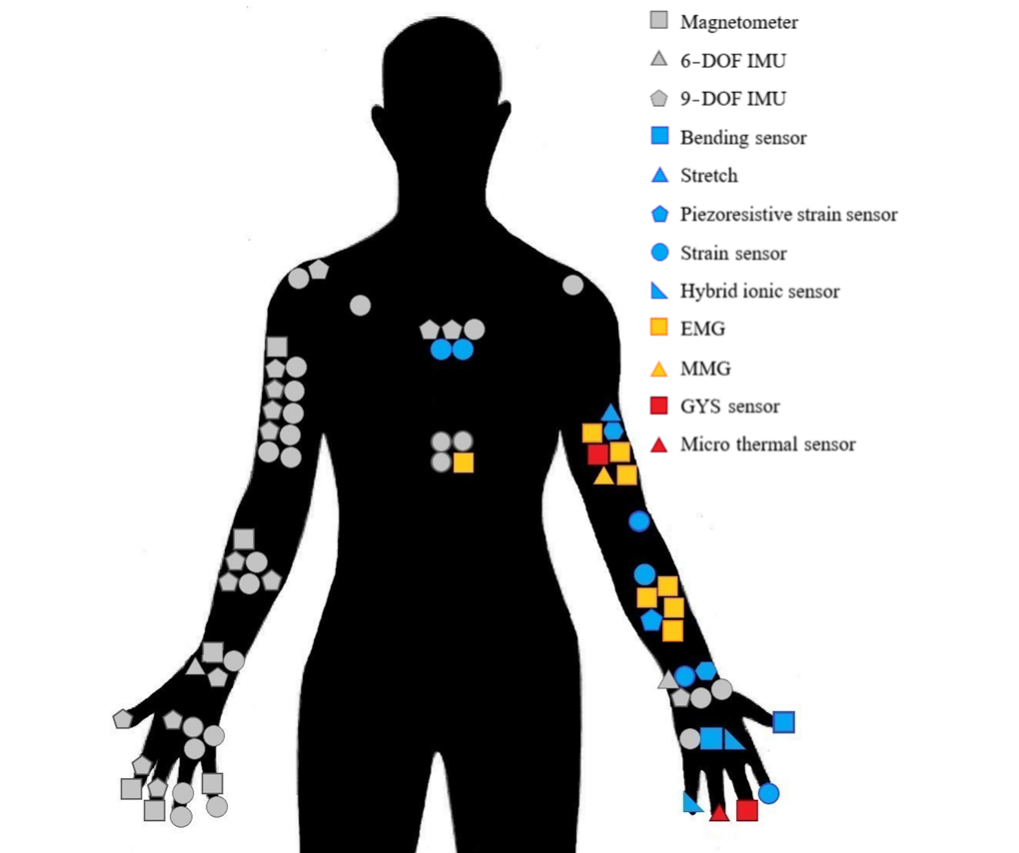
Infograph of sensor placement. This model summarizes the placement of sensors on the upper body; however, the overall number of sensors or whether they are placed on the right or left hand has not been considered. DOF: degrees of freedom; EMG: electromyography; GYS: graphene thin-film yarn sensor; IMU: inertial measurement unit; MMG: mechanomyography.

The placement of each sensor can vary greatly, including the core of the body, across the arm, and on the fingers, according to the intended application. Table 4 provides information about the number and placement of sensors, and Figure 3 graphically shows their distribution for the studies examined in this paper.

**Table 4:** Studies that used sensors in each upper limb location.

Location	Studies	Number of studies
Scapula	[21,28]	2
Torso	[19,24,28]	3
Sternum	[14,20,21,34]	4
Shoulder	[21,24,35]	3
Upper arm	[13,14,18-21,23,26,27,31,34,35,39]	13
Forearm	[14,18-21,23,27,33,34,36,37,39]	13
Wrist	[14,16-18,20,22,23,25,26,29,31,35]	12
Hand	[15,16,20,21,23,30,32,37]	8
Thumb	[28,30,32]	3

Among the 27 studies, 13 [13,14,18-21,23,26,27,31,34,35,39] involved sensor placement in the upper arm, 12 [14,16-18,20,22,23,25,26,29,31,35] involved placement on the wrist, and 13 [14,18-21,23,27,33,34,36,37,39] involved placement on the forearm. Many studies focused on finger movement monitoring. Sensors were placed on the hand in 8 studies [15,16,20,21,23,30,32,37], on the thumb in 3 studies [28,30,32], and on the shoulder in 3 studies [21,24,35]. In some cases, complex body posture and position were monitored. Sensors were placed at the sternum in 4 papers [14,20,21,34], at the torso in 3 papers [19,24,28], and at the scapula in 2 papers [21,28].

#### Placement Method and Feasibility of Sensors

One of the key factors considered for the development of upper limb wearable devices is the feasibility and wearability of the sensors, as in some cases, long-term monitoring is required. Consequently, the design of each system must be examined because it can greatly influence the feasibility of the system. From the reviewed papers, wearable systems can be classified into 4 categories, according to the attachment method of the sensors: (1) hook and loop straps (Velcro straps) and fastened straps; (2) bands; (3) adhesive bonding; and (4) other methods.

With regard to the methods used for placing the sensors, 13 out of the 27 studies [14,16,19,21,24-28,30,34,35,39] selected hook and loop straps. More specifically, 4 studies [26,31,34,39] used only a Velcro strap, which is a fastener that adheres to itself. Straps are generally a preferred type of placement method as they can be placed in convenient places on the arm [27] and they are simple, lightweight, and easy to use [25]. Clip-on straps have also been developed, which are flexible, and they allow inertial sensors to be placed on the fingers [16]. Furthermore, 5 studies [19,21,24,28,35] used a combination of hook and loop straps. Medical tape with 3D-printed flexible straps [21] or Velcro straps [35] were designed for upper limb assessment and rehabilitation of stroke patients, respectively. The former application was characterized by participants as being “comfortable to wear;” however, 3 of them reported impedance to grasp because of the finger sensors. In the latter, the design was a major issue as there is still work in the development of the sensors and their alignment. Straps along with direct winding of sensors in the armband [19] were also used as a method of placement; however, there is no discussion about the feasibility of the system. Another way to improve the feasibility of wearability is by designing a vest where different garment parts are attached with Velcro straps [24]. A zipped vest of soft material was developed, which makes it easier for patients to put on and take off. The precision of the sensor is guaranteed by a predefined position, and it is sewn by coated conductive yarn on a soft elastic strap with a Velcro strap fastened at the end. Overall, this system is perceived as highly usable, and the patients were motivated to train with it. Similarly, a garment embedded with smart textiles, conductive points, yarns, and sensors attached with elastic Velcro straps was also introduced [28]. This system is adjustable and more precise as sensors are placed in different positions, and it was rated as having high usability by users because it resembles everyday clothing in appearance and comfort while accurately tracking posture. Finally, flexible resin straps were also used [14] for attaching the housing cases of the sensors; however, the wearability of the system was not characterized.

Stretchable bands have also been designed [18] as they provide convenience and comfort around the arm without disturbing the subject’s movement [20]. Furthermore, for hand and finger tracking, wristbands and rings were developed [16], and they were attached to the wrist and finger, respectively; however, the feasibility of the application was not discussed. For tracking the forearm, a study [36] used an armband to secure the IMU position; thus, the surface EMG acquisition remained the same for all patients. Feasibility was also not examined for this system.

Another method of attaching sensors to the body is adhesive bonding [15,23,32,37]. In this case, conductive sensors are included in the fabric and support wearable technologies. Their main function is to sense the physical movement of the arm and then transform it into electrical signals. Assessment of hand movement is necessary when evaluating hand function, and many glove-sensing systems lack rotational observability, hand orientation estimation, and user customization. Sensors can be mounted on a double-sided adhesive tape [23] as well as on a polyamide/elastane-fabricated glove [32], which consists of multiple printed circuit board strings that are attached to each finger segment. However, the feasibility of the system was not specified. Skin-friendly tape that attaches individual sensor strips adhesively to the finger segments and a silicon fixture that attaches the base unit of the system to the back of the hand make the system compact and portable. The sensor strips can be removed and replaced, thus increasing the flexibility in different therapy settings and different hand sizes, which makes the system more practical and easier to maintain [37]. A waterborne adhesive is an alternative method of attaching sensors to the hands as the system is fully integrated, but the result is not considered very feasible [15].

Regular elastic fabrics have also been used [33], where a prototype provided an acceptable comfort level and could be adapted to target users with different figures. A commercial elastane-based fitness shirt with elasticity was also used as a “vest,” where sensors were attached by polychloroprene-based adhesive [38]. Comfort and elasticity were the key requirements for the base shirt to conform with the shape of the body of the user.

From the papers included in this review, only 1 study was conducted by using direct winding (the only placement method for attaching sensors to the body) [13]. GYSs were attached by direct winding on varied portions of the human body for monitoring different movements such as finger bending and muscle contraction and relaxation. A new direct-wearing mode, where the GYS can be directly attached to the skin, was introduced, which resulted in the improvement of sensing accuracy. Moreover, flexible fabric straps made of Lycra have been partially sewn on a glove structure [31] to fix the sensor in an optimal position. The feasibility and flexibility of this application are not discussed, and thus, the usability of the system cannot be concluded. A wearable device for tracking the daily use of the wrist and a finger was developed by designing a watch-like enclosure that is worn on the wrist with a small neodymium magnetic ring on the index finger [29]. This design is described as “nonobtrusive” as the ring provides a reliable wireless signal without a power source, and thus, the need for bulky wiring or a battery is eliminated. Because of its design, the “Manumeter” is described as “socially acceptable,” and it can be worn for long durations. In a more recent study [22], a newer version of the “Manumeter” was developed, where a jewelry-like device was fastened to the wrist with a band to monitor its movement. For finger movement, an ipsilateral finger ring was placed on the index finger. However, feasibility was not discussed.

### Signal Processing Techniques and Extracted Features

Wearable upper limb devices provide important information about limb motion through motion analysis. Different signal processing techniques were used, and features were extracted to provide feedback to end users or therapists for better data assessment and interpretation of movement.

Signal processing is essential for such devices to reduce noise signals, transform data, and extract meaningful motion features. A variety of filters are used depending on the data provided by the sensors and the desired output of the system. In 13 studies [14,16-22,26,29,31,32,35], filters were used to remove noise from sensor signals. More specifically, the Kalman filter was used in 2 studies [20,32], which is an algorithm that uses observed measurements over time to produce estimated unknown variables that tend to be more accurate by estimating a joint probability distribution over the variable of each timeframe.

In 3 studies [18,21,32], the Butterworth filter was used to minimize low-frequency noise and high-frequency interferences. Another method to extract data from upper limb devices involves the infinite impulse response filter (IIR) [14,26]. This filter is digital with infinite impulse response and can be designed as a low-pass or high-pass filter. The Madgwick filter is an algorithm that reduces integration errors by the magnetometer and accelerometer, and it was used in 1 study [16]. A combination of 2 of the aforementioned filters was implemented in 1 study [21]. The accelerometer and gyroscope data were low-pass filtered using the Butterworth filter and then passed through the Madgwick filter to integrate the drift of the angular velocity. A complementary filter was implemented in 1 study [31] to reduce the integration drift by providing correct data on the orientation and position of each upper limb segment. More recently, research has been conducted on machine learning (ML) techniques to assist with predicting motion and improving tracking accuracy. For example, the Block Sparse Bayesian Learning (BSBL) algorithm was used in 1 study to reconstruct the accelerometer signal from compressed data [27]. Little et al [19], on the other hand, compared 10 different ML algorithms to predict angle trajectories through the fusion of physiological and kinematic features.

Another important feature of wearable devices is the sampling rates of the sensors, as they determine the quality of the captured data, providing a better understanding of upper limb motion. High sampling rates provide more precise data acquisition; however, battery life is significantly reduced as power consumption is increased. More specifically, in the revised studies, the smallest sampling rate was mentioned in the research conducted by Tolvanen et al [23], where a pressure sensitivity test for light finger touch by a stretch sensor was performed. In contrast, the highest sampling rate was at 2000 Hz in a paper by Pregnolato et al [36], where hand movements of stroke patients for rehabilitation were captured by a 9-DOF IMU with a surface EMG sensor. While both studies provide valuable insights into their respective applications, the battery life was not mentioned. With regard to the battery life, the shortest battery duration was observed in a study by Gu et al [15], where 10 hydrogel-elastomer hybrid ionic sensors were used to capture hand motion, joint bending, hand posture, and gesture. The battery life in this study was 1-2 hours, with an approximate sampling rate of 333.33 Hz, attributed to a 3.3-V rechargeable Li-ion battery. In contrast, the longest battery life was reported by Friedman et al [29], with a duration of 21.5 hours. Power was delivered to 2 triaxial magnetometers and 1 accelerometer to monitor wrist and hand movements, and the sensors were powered by a 3.7-V 450-mAh lithium polymer battery.

The relationship between battery size and sampling rate is crucial as it can affect the efficiency of the wearable sensors. Specifically, in the study conducted by Lee et al [17], a 170-mAh battery (sized 1-2 cm) was used to power an accelerometer with a sampling rate of 67 Hz, and the battery life was approximately 6 hours. In contrast, Wang et al [24] employed a larger 10-cm 3-V battery to power two 9-DOF IMUs with a sampling rate of 50 Hz. This variety of sampling rates and data acquisition reflects the diverse design choices between consumption and device portability in wearable sensor technology.

### Type of Feedback and Accuracy of the System

Feedback is a key feature of the rehabilitation process and motion tracking as it provides important and meaningful information to therapists to interpret the performance of the system. Moreover, it plays an important role in informing patients of their progress, which leads to better recovery chances. The majority of studies (23/27) [13-23,25-27,29-35,37,38] used visual feedback. Three studies provided visual and auditory information [24,36] and haptic feedback along with visual and auditory information [24,28], and 1 study [39] provided only haptic feedback.

More specifically, visual feedback provides therapists and users with more precise information about their tasks and training instructions to achieve the desired position through direct visual analysis of the movement. Haptic feedback is provided by vibrotactile actuators, and small transducers are designed to optimize skin response to vibration. The vibrotactile actuators must have a minimum critical distance for their vibration to be identifiable, and the subjects of the experiment had to rely exclusively on it to accurately rehabilitate their posture and eventually regain their lost muscular abilities. Wearable devices that used a combination of feedback approaches (visual, audio, and haptic) provided an objective outcome that contributed to increasing the effectiveness of training. Moreover, these approaches provide support to therapists, giving them additional information about the patients’ motions, and lastly, the quality of training is improved by detailed feedback signals.

Accuracy of system measurements is necessary for the effectiveness of proposed wearable devices as it plays a vital role in the interpretation of output data and the extraction of user features. Consequently, feedback should be quick enough to improve the operator’s performance in terms of reducing mental effort and informing therapists about movement characteristics. One of the most common ways to present the accuracy of the system and evaluate position and velocity based on a kinematic model is through the calculation of the mean error between the bending joint angle of the user and the proposed validated algorithm with good accuracy, which is used as a reference, under different speeds and magnitudes [14,16,17,19,22,24,25,27,29-37]. Moreover, a statistical analysis is conducted by calculating the “root mean square deviation” (RMSD) or “root mean square error” (RMSE), correlation coefficient (CC), mean absolute error (MAE), and *P* value. In some papers, accuracy was either not measured or the circumstances under which accuracy was calculated were not mentioned [13,15,18,20,21,23,26,28,35,38,39].

## Discussion

### Principal Findings

This paper provides a systematic literature review of upper limb wearable device technologies that were developed in the past decade. The screening of papers from 4 different electronic libraries yielded a total of 27 relevant papers that were included for analysis. The papers were classified into 2 major categories, according to the purpose of the study: clinical motion tracking and rehabilitation. The analysis of the findings suggests that upper limb motion is most often tracked using inertial sensors owing to their accuracy, compact size, and effectiveness in assessing range of motion (ROM). Advanced data processing techniques, such as Kalman and Madgwick filters, were used for data fusion, ensuring data accuracy. One of the key elements of wearable technologies is usability, which can be affected by how wearables are placed on a user, their form factor, and their energy consumption. Even though most studies used straps for placing sensors, the other characteristics have greater variability, with no clear consensus among the research community.

The papers were categorized according to the type of study (controlled and uncontrolled). Most of them were uncontrolled studies, which may lead to bias because of the absence of randomly selected control groups and a comparison between them.

The goal of upper limb wearable devices is to provide assistance to therapists and researchers through either monitoring upper limb function for clinical assessment or aiding rehabilitation training and reducing the recovery time [40]. Overall, the developed wearable devices can positively influence the motivation of users, while in the case of rehabilitation, patients can undergo treatment at home by aiming for a high level of independence [41].

Various sensing technologies were used for tracking upper limb motion. Inertial sensors tend to be the most used sensors as they are used to estimate joint angles of the upper limbs. These technologies provide data accuracy, and because of their size, they are used to monitor and provide feedback to patients and therapists on ROM and rehabilitation performance. However, the placement method needs to be considered owing to its essential role in the ROM assessment, as the interpretation of data influences the development of rehabilitation treatment. Moreover, a flexible and well-fitted design can improve signal quality and reduce measurement noise [42]. Consequently, the majority of devices were attached to the body with straps (mostly Velcro straps), as they provide flexibility and great strength (eliminating data bias) and require low maintenance. The hold that Velcro straps provide can be generally characterized as “firm.” Although this placement method is easy and noninvasive [39] and there is no need for external cameras, emitters, or markers [31], the feasibility of the system is not guaranteed for an extended period [25,39]. Similarly, clip-on straps can make the system modular in many aspects. This design can be adopted regardless of hand dimensions and the presence of deformities or inflammation. These straps are manufactured by using a stretchable and flexible material that yields extra comfort and causes minimum disruptions to movement [16].

The placement of wearable sensors significantly impacts their performance, user acceptance, and engineering demands. As sensor technology progresses from wearable to implantable and ingestible forms, challenges arise across regulatory, technical, and translational domains. Misplacement or misalignment of wearable sensors can reduce classifier accuracy; however, some approaches have maintained precision (97%) and recall (98%) at high levels during movement classification [7,9].

Despite the utility of straps, challenges remain in optimal sensor placement for maintaining user comfort and mobility and managing interference between sensors. Overcoming these challenges includes performing comprehensive user studies and data analyses to determine the best sensor placement, as well as designing an ergonomic and adaptable sensor housing for enhancing user comfort [43]. Thus, the ergonomic aspects of the system, such as dimension, weight, and undesirable contact, should be considered to accommodate various hand sizes and deformations [16]. Recent innovations have focused on achieving body compliance, ensuring comfort for the wearer, and maintaining accurate sensing performance [43].

Furthermore, some studies used smart textiles or e-textiles with embedded sensors as the sensing technology, which provided great feasibility regarding wearability. The use of textiles in health care and wellness applications has increased over the last few years and is expected to grow further in the future [41]. The primary benefit of using textile-based electrodes is that there is no direct contact with the skin, and this prevents problems like allergy and skin irritation [44]. Nevertheless, additional research needs to be conducted for integrating accuracy, improving usability, and implementing clinical validation. We expect that the benefits provided by textiles, especially related to ease of use and flexibility, and the advances in technology will make them essential for tracking motion and muscle activity and improving rehabilitation outcomes [44,45].

Wearable devices capture upper limb motion through data acquisition and processing. The examined studies used various measurement methods to record data such as body segment posture, amount of use, and ROM. Undoubtedly, data processing is necessary for developing wearable devices as the signals captured have to be interpreted. The most common filters are Butterworth, low-pass, and band-pass, which are used to weaken potential high-frequency noise in the accelerometer and gyroscope. Moreover, Kalman, Madgwick, and complementary filters are used to fuse the sensor readings and overcome potential biomechanical constraints. These algorithms combine the sensor readings to indicate the rotation and orientation of the arm; however, they tend to require high computational power.

Sampling rates across the analyzed studies varied significantly and ranged from as low as 2 Hz for pressure-sensing technologies to as high as 2000 Hz for motion-sensing applications. Balancing high sampling rates and energy efficiency remains a challenge for upper limb wearable devices, as higher rates enhance tracking precision but can reduce battery life. The wide range of rates indicates the adaptability and flexibility of the sensors used for upper limb applications, as they capture muscle movements as well as rapid motion movements with precision. However, the selection of the right battery to support the desired sampling rates remains a challenge, and there is a need for careful selection to ensure prolonged and uninterrupted data acquisition.

Different techniques are being developed that aim to improve energy efficiency and extend the battery life of wearable devices. For example, compressed sensing [46] allows the reduction of the sampling rate of a signal, which can then be transmitted using a compressed sparse representation and reconstructed with minimal loss compared to the original signal [47,48]. Another algorithm that can be used with time series data, such as motion and muscle data, and can improve energy efficiency is change point detection [49]. The algorithm is used to detect the point when a signal changes and is often used to assist with action recognition [50]. Therefore, by detecting the time point when a signal changes, the sampling rate can be adapted and energy consumption can be reduced during nonrelevant activities (eg, transition between motions, resting, etc) [49]. At the same time, recently, research has been conducted on extending battery life through energy harvesting [51]. The process relies on capturing energy either directly from the human body, including motion [43], or from the environment and converting it to power.

Feedback plays a crucial role in the overall system performance as it provides useful information to therapists and researchers and affects therapy outcomes by influencing motivation. Feedback is given frequently to users who are less proficient or whose posture needs to be improved. This is beneficial for future applications as users should not rely on external feedback, but instead follow their intrinsic feedback mechanics. Visual information on a computer or smartphone/tablet is usually provided, which is very useful, especially for systems that are remotely monitored. Ultimately, the main aim of feedback is to help users improve their performance while providing useful data to therapists for better information processing and future reference.

The improved computation power of electronics and advances in ML algorithms have increased the use of these algorithms for not only motion trajectory prediction [52] but also assessing motion quality and providing relevant feedback during rehabilitation [53]. Using ML with wearable devices through Tiny Machine Learning (TinyML) has gained popularity recently; however, many challenges remain [54]. Further research in this area can revolutionize the use of wearable technology in health care applications by providing greater accuracy, and improved and more personalized feedback. Feedback can also be further reinforced using haptic devices [55]. However, current haptic devices can be cumbersome, and more portable devices need to be developed to make such technologies easy to use in a clinical setting [56].

### Limitations and Conclusion

In this review, an effort was made to cover studies related to upper limb wearable devices, including study purpose, sensing technology, feasibility and wearability of the system, sensor placement, methodology, and feedback received. However, because of the variety of studies conducted in this area, every aspect could not be covered, and hence, a summary was provided for aspects with relatively more research. Limitations, such as the positioning, number, and possible disruption of sensors, are challenges that need to be overcome as they affect the limb’s computed trajectory. Moreover, when a magnetometer is used, ferromagnetic materials can affect its calibration, and the data may not be transmitted with accuracy. Additionally, the lack of uniformity of battery specifications (compared to sampling rates) and sensor specifications highlights the challenges in comparisons between these characteristics and standardized data in this field. Although the studies reviewed indicated a positive influence regarding the motivation of users, more clinical trials need to be conducted, as they are important to assess the effectiveness of the system. Another limitation is the search strategy employed in this review. The search was performed in only 4 specific databases: ACM Digital Library, IEEE Xplore, PubMed, and ScienceDirect. The search may have excluded relevant papers, and some studies might have been overlooked. The low number of studies analyzed might not fully capture the diversity of the current research in the field, which limits the comprehensiveness of the review.

Future studies should aim to reduce the weight and dimensions of the system and increase the sampling rate, which can facilitate quick motion tracking with high accuracy. Additionally, efforts should be made to fabricate upper limb devices that are more flexible, powerful, and compact. The progress of ML algorithms would be beneficial, particularly in IMU- and EMG-based devices, as the rehabilitation process is automatically guided in real-life settings while being able to provide remote intervention [55]. Moreover, researchers should focus on the further development of feedback as it should be more adaptable and provide more options to users and therapists. In addition, long-term clinical trials are essential for establishing the effectiveness of wearable devices in real-world rehabilitation settings. These trials should focus on larger and more diverse patient groups to better establish the system’s efficacy. Moreover, in broader health care frameworks, standardized protocols and consistent measurement methods should be developed to ensure the results are accurately compared, thus ensuring the reliability of the system. Behavioral measurements are also important, especially in training sessions. In conclusion, future research should focus on integrating sensors and improving usability and feasibility as upper limb wearable devices are predictably becoming powerful tools, enabling innovative rehabilitation treatments while improving the quality of health care.

## Appendix

Multimedia Appendix 1 PRISMA (Preferred Reporting Items for Systematic Reviews and Meta-Analyses) checklist.

## Abbreviations

DOF: degrees of freedom
EMG: electromyography
GYS: graphene thin-film yarn sensor
IMU: inertial measurement unit
ML: machine learning
MMG: mechanomyography
ROM: range of motion

## Data Availability

All data generated or analyzed during this study are included in the published articles mentioned in the review.
