# Supplementary material for: Tracking Upper Limb Motion via Wearable Solutions: Systematic Review of Research From 2011 to 2023
Source: J Med Internet Res. 2024 Dec 23;26:e51994. doi: 10.2196/51994 (PMC11704657; doi:10.2196/51994)
Supplement: Multimedia Appendix 1 [file jmir_v26i1e51994_app1.pdf]

Section/topic	#	Checklist item	Location(s) Reported
**INFORMATION SOURCES AND METHODS**			
Database name	1	Name each individual database searched, stating the platform for each.	3
Multi-database searching	2	If databases were searched simultaneously on a single platform, state the name of the platform, listing all of the databases searched.	N/A
Study registries	3	List any study registries searched.	4
Online resources and browsing	4	Describe any online or print source purposefully searched or browsed (e.g., tables of contents, print conference proceedings, web sites), and how this was done.	3
Citation searching	5	Indicate whether cited references or citing references were examined, and describe any methods used for locating cited/citing references (e.g., browsing reference lists, using a citation index, setting up email alerts for references citing included studies).	5-6
Contacts	6	Indicate whether additional studies or data were sought by contacting authors, experts, manufacturers, or others.	N/A
Other methods	7	Describe any additional information sources or search methods used.	4
**SEARCH STRATEGIES**			
Full search strategies	8	Include the search strategies for each database and information source, copied and pasted exactly as run.	3-4
Limits and restrictions	9	Specify that no limits were used, or describe any limits or restrictions applied to a search (e.g., date or time period, language, study design) and provide justification for their use.	4-5
Search filters	10	Indicate whether published search filters were used (as originally designed or modified), and if so, cite the filter(s) used.	4
Prior work	11	Indicate when search strategies from other literature reviews were adapted or reused for a substantive part or all of the search, citing the previous review(s).	N/A
Updates	12	Report the methods used to update the search(es) (e.g., rerunning searches, email alerts).	N/A
Dates of searches	13	For each search strategy, provide the date when the last search occurred.	N/A
**PEER REVIEW**			
Peer review	14	Describe any search peer review process.	N/A
**MANAGING RECORDS**			
Total Records	15	Document the total number of records identified from each database and other information sources.	3-4
Deduplication	16	Describe the processes and any software used to deduplicate records from multiple database searches and other information sources.	4-6

PRISMA-S: An Extension to the PRISMA Statement for Reporting Literature Searches in Systematic Reviews
Rethlefsen ML, Kirtley S, Waffenschmidt S, Ayala AP, Moher D, Page MJ, Koffel JB, PRISMA-S Group.
Last updated February 27, 2020.
